# Real-time PCR/MCA assay using fluorescence resonance energy transfer for the genotyping of resistance related DHPS-540 mutations in *Plasmodium falciparum*

**DOI:** 10.1186/1475-2875-7-48

**Published:** 2008-03-17

**Authors:** Petra F Mens, Chantal van Overmeir, Maryline Bonnet, Jean-Claude Dujardin, Umberto d'Alessandro

**Affiliations:** 1 Prince Leopold Institute of Tropical Medicine, Department of Parasitology, Nationalestraat 155, B2000, Antwerp, Belgium; 2 Koninklijk Instituut voor de Tropen/Royal Tropical Institute, KIT Biomedical Research, Meibergdreef 39, 1105 AZ Amsterdam, The Netherlands; 3 Epicentre, 8 rue St. Sabin, 75011 Paris, France

## Abstract

**Background:**

Sulphadoxine-pyrimethamine has been abandoned as first- or second-line treatment by most African malaria endemic countries in favour of artemisinin-based combination treatments, but the drug is still used as intermittent preventive treatment during pregnancy. However, resistance to sulphadoxine-pyrimethamine has been increasing in the past few years and, although the link between molecular markers and treatment failure has not been firmly established, at least for pregnant women, it is important to monitor such markers.

**Methods:**

This paper reports a novel sensitive, semi-quantitative and specific real-time PCR and melting curve analysis (MCA) assay using fluorescence resonance energy transfer (FRET) for the detection of DHPS-540, an important predictor for SP resistance. FRET/MCA was evaluated using 78 clinical samples from malaria patients and compared to PCR-RFLP.

**Results:**

Sixty-two samples were in perfect agreement between both assays. One sample showed a small wild type signal with FRET/MCA that indicates a polyclonal infection. Four samples were not able to generate enough material in both assays to distinguish mutant from wild-type infection, six samples gave no signal in PCR-RFLP and five samples gave no amplification in FRET/MCA.

**Conclusion:**

FRET/MCA is an effective tool for the identification of SNPs in drug studies and epidemiological surveys on resistance markers in general and DHPS-540 mutation in particular.

## Background

Malaria control is hampered by increasing spread of drug resistance [1]. Although sulphadoxine-pyrimethamine (SP) has been abandoned as first- or second-line treatment by most African malaria endemic countries in favour of artemisinin-based combination treatments (ACT), it is still used as intermittent preventive treatment during pregnancy (IPTp) [2-4]. However, SP resistance has been on the rise in the past few years and, although the link between molecular markers and treatment failure has not been firmly established, at least for pregnant women, these markers should be monitored. Pyrimethamine binds to malarial dihydrofolate reductase (DHFR) and inhibits its enzymatic activity. Sulphadoxine competes with a natural substrate of dihydropteorate synthase (DHPS) and consequently inhibits the enzyme. Resistance to these drugs has been associated with single nucleotide polymorphisms (SNP) in the genes encoding DHFR and DHPS [5-8]. One of the SNPs that is associated with SP drug resistance is the Lys → Glu substitution in codon 540 of the DHPS gene. Although there is conflicting evidence about the role of the *dhps *Glu-540 mutation it is considered a strong predictor for SP treatment failure. In some studies that describe treatment fialure no DHPS gene is found but in other studies in for example Uganda the glu-DHPS mutation was found to be the strongest independent predictor of treatment failure [8,9]. In addition they found that other important mutations such as the *dhfr *Arg-59 mutation were only predictive of treatment failure in the presence of the *dhps *Glu-540 mutation [9].

**Figure 1:**
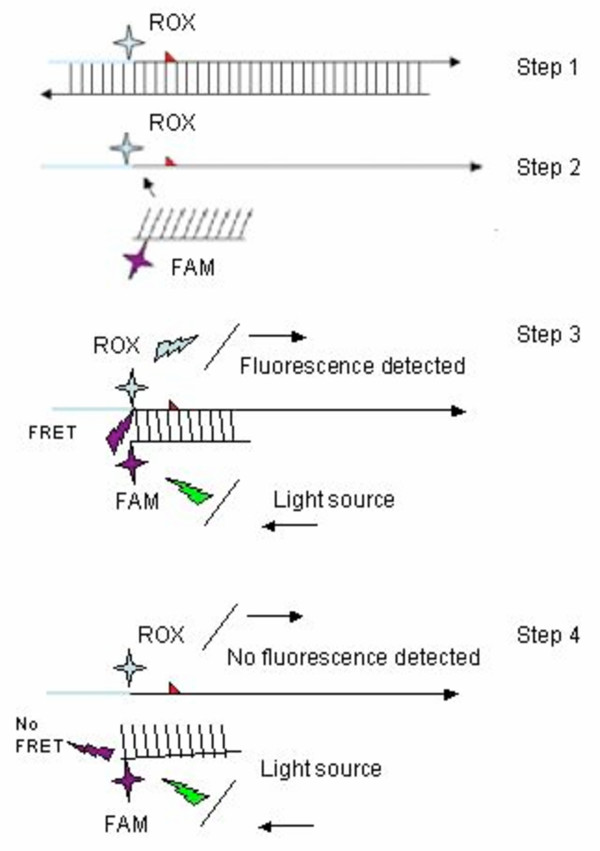
**Schematic representation of FRET assay**. Step 1. A PCR reaction results in amplification of a 169 bp fragment with an incorporated ROX fluorophore attached to the forward primer. Step 2. After amplification, the FAM-labelled probe is added to the reaction and during initial denaturation hybridizes to the amplicon. Step 3. The two fluorophores are now in close proximity of each other and energy from the excited donor is transferred to the acceptor generating the FRET signal. Step 4. The increase of temperature during melt curve analysis leads, at a specific temperature, to the dissociation of the probe from the amplicon. When the probe is dissociated transfer of energy is lost and therefore no FRET signal can be observed.

In most countries chloroquine (CQ) has been, for many years, the first line drug for malaria treatment. Unfortunately, resistance to this drug is nowadays widespread. However, in areas where drug pressure has been removed, the molecular markers linked to CQ drug resistance seem to disappear [10,11], indicating that local parasites are again sensitive to this drug. It is, therefore, important to have the means for monitoring drug resistance and understand its spread as this may be important in design strategies to prevent the selection of drug resistant parasites [8,12]. The spread of point mutations linked to drug resistance could be monitored by carrying out regular cross sectional surveys and then genotyping the samples from infected people. Molecular tools for the detection and identification of these SNPs, such as Amplification Refractory Mutation System PCR (ARMS-PCR) or Restriction Fragment Length Polymorphism PCR (RFLP-PCR), are available [13,14]. Although RFLP-PCR is widely used to identify SNPs, it is a time consuming, low throughput and labour-intensive technique, that is not able to quantify different genotypes in a polyclonal infection and is prone to DNA contamination [15,16]. Fluorogenic assays can be a good alternative for the detection of SNP mutations conferring drug resistance [16,17]. PCR in combination with fluorescence resonance energy transfer (FRET), which uses two fluorophores that are brought in close proximity after hybridization, can detect point mutations in DHFR gene linked to pyrimethamine resistance[18]. In short, the principle of FRET is as follows: during PCR, a primer labeled with fluorophore which are called "donor" hybridizes to the formed amplicon. After PCR, an additional probe with a fluorophore "the acceptor" is added tot the reaction and, during denaturation, anneals to the template. The fluorescence emitted from the donor is then absorbed by the acceptor, resulting in fluorescence emission by the acceptor, FRET, which can be detected. When this reaction is followed by melt curve analysis (MCA), dissociation of the fluorophores and consequently loss of FRET signal occurs. This depends on the thermodynamic stability of the probes, allows the discrimination between total complementary probe target (wild-type samples) and mismatched probe target combinations (mutant samples) (Figure 1) [19].

This paper describes a new FRET/MCA assay with a fluorogenic primer/probe design in combination with MCA, which enables: 1) the identification of the genotype (wild type or Lys → Glu substitution) in codon 540 of the DHPS gene of *Plasmodium falciparum *that is highly associated with sulphadoxine resistance; 2) the quantification of the different genotypes in a polyclonal infection.

## Materials and methods

### Samples

Seventy-eight field samples were obtained in the context of a larger study, in collaboration with Epicentre (Paris, France) on the therapeutic effects of anti-malarial drugs for the treatment of falciparum malaria in the Democratic Republic of Congo [13]. The samples were collected in Boende, Kilwa and Kisangani during the malaria transmission season between September 2003 and March 2004, after obtaining ethical approval from the national health authorities and the research ethics committee of Prince Leopold Institute of Tropical Medicine, Antwerp (ITMA). The human experimentation guidelines of ITMA were followed.

After obtaining informed consent, finger-prick blood samples were collected at initial diagnosis onto a 3 M Whatman filter paper and stored at room temperature in an individual zip-lock polyethylene bag until further DNA extraction.

Parasitaemia was determined from Giemsa-stained thick smears by counting the number of parasites against 200 leucocytes assuming that there were 8,000 leucocytes/μl blood. Plasmids containing a DHPS insert of a known genotype, i.e. PS-Mali (wild type for codon 540) and PS-Peru (mutant Lys → Glu substitution), were obtained from the Malaria Research and Reference Reagent Resource Center, Division of Microbiology and Infectious Diseases, NIAID, NIH, deposited by C. Plowe. To determine the capability of the assay to discriminate between mixed infections, artificial mixes of these clones were made in rations of 1:9/9:1. The estimated percentage of the genotypes in the mixture are represented by the area underneath the peak for that genotype and automatically calculated by the intergtrated software.

As a reference, DHPS genotyping for DHPS codon 540 was done by PCR followed by restriction digestion as previously described [12]. The products were visualized by a UV light transilluminator after running the products for three hours at 50 volt on a 3% ethidium bromide stained agarose gel. PS-Peru and PS-Mali were used as controls.

### Primers and probe design

Nested PCR was used to amplify enough bulk template for optimization with specific primers flanking the codon 540 of the DHPS gene (710 bp); forward primer: 5'-AACCTAAACGTGCTGTTCAA-3'; reverse primer: 5'-AATTGTGTGATTTGTCCACAA-3' previously published [12]. Secondary PCR primers amplifying a 169 bp product, (forward primer: 5'-ATGCATAAAAGAGGAAATACACATAC-3' and reverse primer: 5'-TCATGTTTCTTCGCAAATCC-3') were selected based on the absence of primer dimers, melting temperature, hairpin and secondary structure configurations and their GC content and analysed with Primer3 [20] and DNA mfold [21]. In order to prevent hairpin formation, a mutation in the forward primer was introduced. The forward primer was labeled with an internal 5-carboxy-x-rhodamine (ROX) at the last thymine (base 24).

All primers were checked for their specificity by a BLAST search [22].

The fluorogenic probe was designed to have the highest difference in melting temperature between the wild type and mutatant strand as calculated by Meltcalc, [23]. The probe 5'-CATAATTTGTTAGTTTATCCA-3'complements the wild type antisense strand of the PCR product and was labeled with 3' 6-carboxyfluorescein (FAM).

### FRET-PCR

A primary PCR was performed before starting the FRET/MCA analysis to increase sensitivity. Each reaction of 25 μl contained 200 μM MgCl, 200 μM dNTP, 1× reaction buffer (Promega), 5 U/μl Taq DNA polymerase (Promega), and 250 nM of forward and reverse primer. To each reaction 3 μl of template was added. For amplification the following protocol was used: 3 min 94°C followed by five cycles of 1 min 94°C, 2 min 45°C and 1 min 72°C. This was followed by 35 cycles of 1 min 94°C, 1 min 45°C and 1 min 72°C and a final step of 10 min 72°C.

Following primary amplification a FRET/MCA PCR was performed. All FRET/MCA assays were performed on a iCycler (BioRad Hercules, USA) and analysis of the results was done by iCyclerIQ optical system software version 3.0a (BioRad Lab inc.). Each reaction of 50 μl contained 1× IQsupermix (BioRad laboratories) and the complete primer/probe set. The target strand to which the probe binds was produced in excess by an asymmetric PCR containing 500 nmol/L ROX labeled forward and 100 nmol/L reverse primer. The probe (160 nmol/L) was added directly after amplification after which the MCA was performed. The total protocol was as follows: 3 min 95°C, 35 cycles of 30 sec 95°C, 1 min 58°C, 1 min 72°C and a final step of 8 min 72°C.

### Melt curve analysis

After amplification the melt curve analysis (MCA) was performed with a 490/20× FAM excitation filter and a 620/30 M ROX emission filter. Immediately after the PCR, probe was added starting at 60°C with 60 repeats of heating for 30 sec with 0.5°C increments. All experiments were performed in triplicate and in addition to DNA template, MilliQ and plasmids PS-Mali and PS-Peru were used as control samples. Well factor errors were subtracted from the post run data by using a 96 well optical plate containing 50 μl 1× external well solution in each well (BioRad Laboratories) before the actual MCA. These factors are used by the system to compensate for any pipetting or system inconsistencies in order to optimize the post-run data analysis.

### Statistical analysis

PCR-RFLP for the DHPS-540 identification was considered as the reference method. All FRET-PCR/MCA results were compared to the results obtained with the PCR-RFLP standard. The agreement between PCR-RFLP and the FRET-PCR/MCA assay was determined by calculating Kappa statistics values with a 95% confidence interval [24]. Kappa statistics is measure of agreement between categorical measurements, in this case the outcome of two diagnostic tests, in which the accuracy of a test is calculated. It is thus an index which compares the observed agreement against that which might be expected by chance. Kappa can be thought of as the chance-corrected proportional agreement, and possible values range from +1 (perfect agreement) via 0 (no agreement above that expected by chance) to -1 (complete disagreement). Kappa values of 0.21 – 0.60 is a moderate, a kappa value of 0.61–0.80 a good and kappa > 0.80 an almost perfect agreement beyond chance.

## Results

### Analytical performance of the assay

**Figure 2:**
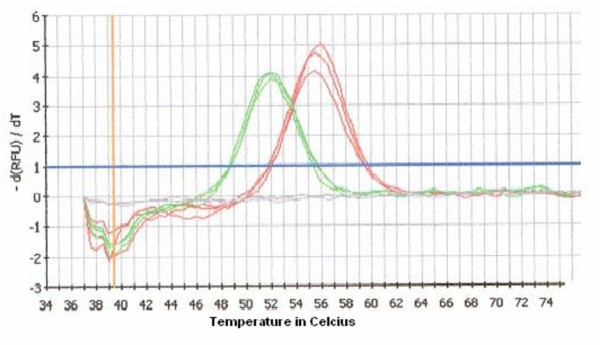
**Output file of the MCA assay with plasmid DNA**. The figure shows the melt curve analysis of wild type clone PS-Mali in triplicate (red curves) resulted in an average Tm of 55.7°C. In green the melt curve profile (in triplicate) of mutant clone PS-Peru is shown with a Tm of of 51.9°C. The change in amount of fluorescence for each probe-template hybrid was plotted against the temperature and its negative derivative appeared as a positive peak. The grey lines represent the negative controles.

**Figure 3:**
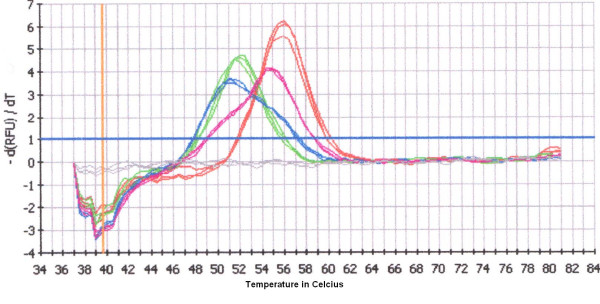
**Identification of a polyclonal infection**. Blood samples from two patients with malaria that have a biclonal infections. The MCA curves of these samples with two peaks represented in blue and purple are shown. The sample represented in purple contains according to the intergrated software a 45% to 55% ratio of mutant and wild type infection respectively. The sample represented in blue shows the opposite pattern and contains 55% to 45% mutant and wild type infection respectively. This can also be seen in the figure by the difference in peak hight. The red curves are the positive controls for wild type strains and the green for mutant strains. The blue horizontal line represents the threshold for background fluorescence, and the curve entirely below shows the results for the negative control (gray).

Predictions made by Meltcalc on the melting temperature (Tm), resulted in stability calculations of the sequence with and without mutation of 54.5°C and 51.0°C, respectively. Analysis with MCA on wild type clone PS-Mali resulted in an average Tm of 55.7°C (n = 15; std = 0.41°C) and of an average Tm of mutant clone PS-Peru of 51.9°C (n = 15; std = 0.32°C) (Figure 2). However, when analysing field samples the average Tm for wild type samples was 51.5°C and 55.0°C for the mutant samples (Figure 3).

Artificial mixed infections of PS-Mali and PS-Peru clones gave observable peaks from mix ratios 2:8 to 8:2, which were also calculated as such by the software. The ratios 1:9 and 9:1 gave no observable second peak after MCA and identified the mixes as being a mono infection of either PS-Mali or PS-Peru.

### Analysis of field samples

In total, 78 microscopy positive samples (range: 16 parasites/μl – 197400 parasites/μl) were analysed with PCR-RFLP as well as with FRET/MCA. PCR RFLP obtained a result in 68 samples. The remaining 10 samples, which were found positive with microscopy (16–46 parasites/μl) showed no amplification with PCR-RFLP. FRET/MCA could classify 69 samples; in the other 9 no MCA results could be obtained. The samples that were negative with MCA and or RFLP were all samples that contained very low parasite numbers as revealed by standard microscopy (<50 parasites/μl) probably leading to an insufficient template amount for PCR. The results of both assays are presented in Table 1.

**Table 1:** Overview of the mutant, wild type and mixed infections of the 78 samples analysed with PCR-RFLP and FRET/MCA.

**PCR-RFLP**	**FRET/MCA**			
	
	**W**	**M**	**W/M**	**N/A**
**W**	**27**	0	0	4
**M**	0	**21**	1	0
**W/M**	0	0	**14**	1
**N/A**	4	1	1	**4**

W = wild type; M = mutant, W/M = mixed infection with wild type and mutant genotype, N/A = no amplification

In the 15 samples identified as a mixed infection by FRET/MCA, five samples had an equal amount of both wild type and mutant parasites. The wild type was dominant in six samples and four samples had the mutant type as dominant polymorphism. The ratios varied between 30%–70% mutant/wild type to 70%–30% wild-type/mutant. One sample showed a mutant infection in PCR-PFLP and a mixed infection with FRET/MCA with a small wild-type peak in addition to a dominant mutant peak.

### Statistical analysis

A high degree of agreement was observed between the FRET/MCA assay and PCR/RFLP in the present study for the identification of mutant or wild-type strains in a sample. The overall kappa value of 0.7828 (95% CI: 0.6719–0.8937) indicates a good agreement beyond chance. The kappa for wild type and mutant mutations is 1 (95% CI: 0.9647–1) and the kappa for the identification of mixed infections is 0.9552 (95% CI: 0.8765–1).

## Discussion

Understanding the mechanisms of drug resistance is of major importance for malaria control. However, good methods to identify drug resistance markers, monitoring spread of resistance and the ability to quantify polymorphic populations in mixed infections is currently lacking. In the light of SP resistance on the one hand and the implementation of IPTp on the other hand, it is becoming very important to carefully examine the presence of resistance markers in the population [1-3,25]. Molecular techniques such as ARMS-PCR and PCR-RFLP are available for the detection of different mutations but have disadvantages such as long processing time and the fact that quantification of mixed infections is not possible [13,15,16]. Although real-time PCR assays able to (semi)-quantify are currently available, they often do not discriminate between single and polyclonal infections [16,17]. This paper describes the development of a semi-quantitative FRET/MCA assay for the detection of SNPs in codon 540 of the DHPS gene of *P. falciparum *able to detect polyclonal infections. The FRET/MCA assay has already successfully been used for the identification of SNPs in several genomes, including several mutations in the DHFR gene of *P. falciparum *[18]. Although this FRET/MCA technique requires a high initial equipment outlay, consisting of a standard PCR cycler equipped with an optical module, and uses expensive reagents such as the flurorophores, which make this technique three times more expensive (7 euro per assay) than RFLP analysis, it offers several advantages over existing methods. Methods such as sequencing are also able to identify SNPs, but require purchase and setting up special sequence facilities and additional sample preparation reagents and steps. The major advantage of the FRET/MCA technique is its ability to screen simultaneously for mutant as well as wild type specimens in one reaction with the use of only one probe. In contrast, two separate enzymatic reactions are required to genotype the specimens with RFLP analysis. The total handling time of a RFLP analysis takes around two days whereas a FRET/MCA analysis can be performed in less than one day. In addition, the FRET/MCA is designed to run in a 96-well format with integrated software to analyse the fluorescence data which makes analysis compared to for example sequencing very easy. In the initial development stage, samples were run in triplicate to verify reproducibility but the high reproducibility and ease of interpretation allows up to 90 samples to be analyzed in a single run. Secondly, if new mutations arise at the probe-amplicon hybrid, these mutations can simply be detected by the FRET/MCA assay. RFLP analysis or Q-PCR methods, in contrast, can only detect resistance that are already known and for which an assay is available.

This study shows that the developed assay is able to correctly identify mutant and wild type samples when compared to PCR-RFLP. Mixed infections can readily be identified with both assays. The assay allows semi-quantitative analysis of the sample and is able to identify the dominant type in a sample with high throughput. One of the tested samples identified a sample with a low concentration of wild type parasites whereas the PCR-RFLP identified this sample as a mutant infection. Although this would suggest higher sensitivity of the assay there were some samples with very low number of parasites that were not able to give a signal. Nevertheless, the assay is as sensitive as PCR-RFLP. When this assay could be combined with the DHFR assay it would allow the rapid and high throughput screening of large number of samples.

## Conclusion

The developed real-time FRET/MCA for the detection of single nucleotide polymorphisms in the region of codon 540 of the DHPS gene of *P. falciparum *is an easy and very specific technique. The assay can be used for the detection, identification and semi-quantitative measurement of SNP not only in single genotype but also mixed genotype populations. The sensitivity and specificity of the assay makes the FRET/MCA an effective tool for the identification of SNP's in drug studies or epidemiological surveys for the determination of the prevalence of resistance markers, more particularly for that of the DHPS-540 mutation.

## Authors' contributions

PFM: Development and design of the FRET/MCA assays, validation of the assay and molecular analysis of samples with FRET/MCA. Analysis and interpretation of the data, drafting and preparing the manuscript. CVO: Analysis of clinical samples with PCR-RFLP and analysis of the data MB: PI in Boende, Congo and responsible for the field trail and collection of the clinical samples JCD: Conception of the study and participation in its design on molecular level. Interpretation of the data, critically reading of the manuscript UDA: Conception of the study and participation in its design on epidemiological level. Interpretation of the data, critically reading of the manuscript. All authors read and approved the final manuscript.
